# The Endothelium Solves Problems That Endothelial Cells Do Not Know Exist

**DOI:** 10.1016/j.tips.2017.01.008

**Published:** 2017-04

**Authors:** John G. McCarron, Matthew D. Lee, Calum Wilson

**Affiliations:** 1 Strathclyde Institute of Pharmacy and Biomedical Sciences, University of Strathclyde, SIPBS Building, 161 Cathedral Street, Glasgow G4 0RE, UK

## Abstract

The endothelium is the single layer of cells that lines the entire cardiovascular system and regulates vascular tone and blood–tissue exchange, recruits blood cells, modulates blood clotting, and determines the formation of new blood vessels. To control each function, the endothelium uses a remarkable sensory capability to continuously monitor vanishingly small changes in the concentrations of many simultaneously arriving extracellular activators that each provides cues to the physiological state. Here we suggest that the extraordinary sensory capabilities of the endothelium do not come from single cells but from the combined activity of a large number of endothelial cells. Each cell has a limited, but distinctive, sensory capacity and shares information with neighbours so that sensing is distributed among cells. Communication of information among connected cells provides system-level sensing substantially greater than the capabilities of any single cell and, as a collective, the endothelium solves sensory problems too complex for any single cell.

## Trends

The endothelium simultaneously senses multiple small-noisy and high-intensity signals arriving from various activators. The endothelium specifically links each of the various activators with particular functions and physiological outputs.

An understanding of the sensitivity of single endothelial cells provides minimal insight into overall sensing by the endothelium.

The endothelium is a heterogeneous collection of cells each with different sensitivities to activators.

Communication of information via short-range network properties determines the endothelium’s sensing capabilities, the effectiveness of information transfer, and the resilience of the endothelium to faults.

The collective sensory capacity of the endothelium is beyond the ability of any single endothelial cell and enables the endothelial collective to efficiently discriminate multiple signals of various magnitudes.

## Features of Endothelial Signalling

The endothelium is the innermost layer of cells lining the entire vascular system and is a sophisticated sensory and signal processing centre that controls virtually every cardiovascular function. The endothelium differs from most sensory systems in that each endothelial cell is capable of detecting several different types of input and can generate several different types of output; most sensory systems detect one input and generate one output. The different outputs permit the endothelium to regulate blood pressure and the rate and distribution of blood flow by determining vascular tone and controlling cell proliferation and migration [1] in the blood vessel wall. The endothelium also acts as a vector for the formation of new blood vessels to determine the distribution of blood flow. Impairment of endothelial function (endothelial dysfunction) in the control of blood vessel activity underlies vascular conditions such as hypertension and atherosclerosis and the blood flow problems that occur in diabetes. The endothelium also controls blood fluidity by providing thrombin inhibitors and receptors for protein C activation to prevent blood clot (thrombus) formation. When vascular injury occurs, endothelial cells stop secreting coagulation and aggregation inhibitors and instead secrete von Willebrand factor (VWF) to initiate platelet aggregation and blood coagulation. Overactive clotting causes significant health problems and may block blood vessels by embolism. Endothelial cells also play key roles in immune and inflammatory reactions by regulating lymphocyte and leucocyte movement into tissues via expression of specific proteins cell adhesion molecules to sites requiring defence or repair 2, 3, 4. Yet another function of the endothelium is control of the separation of tissue from blood components within the blood vessel. The endothelium controls blood–tissue separation by determining vascular permeability through a size-selective sieving process controlled by the gaps between cells. While this process is normally tightly controlled, excessive opening of intercellular gaps alters vascular leakage and can lead to the formation of protein-rich oedema in tissue – a hallmark of inflammation. If untreated, inflammation of this type can cause fatal diseases, such as acute respiratory distress syndrome.

Underlying the control of many of these functions is the endothelium’s ability to detect and respond to hundreds of different stimuli. The endothelium receives and integrates information from hormones, neurotransmitters, endothelial cells, pericytes, smooth muscle cells, various blood cells, viral or bacterial infection, proinflammatory cytokines, and oxygen tension. The endothelium is also sensitive to several types of mechanical signals such as those derived from blood pressure and the flow of blood.

To sense each of these signals, the endothelium uses a multitude of receptors to constantly sample the extracellular environment. The endothelium must accurately detect the signals and correctly relay messages so that information is not lost. However, many chemical stimuli fluctuate around basal concentrations creating a small signal on a noisy baseline and resulting in a difficult detection problem. This detection problem is intensified by the number (minimally, tens) of simultaneously arriving messages. Accurately detecting signals barely above and even contained within basal noise values is challenging in all biological systems. The mechanism by which the endothelium detects multiple noisy signals while remaining responsive to high-intensity activation is central to the endothelium’s function but is unresolved.

**Figure 1:**
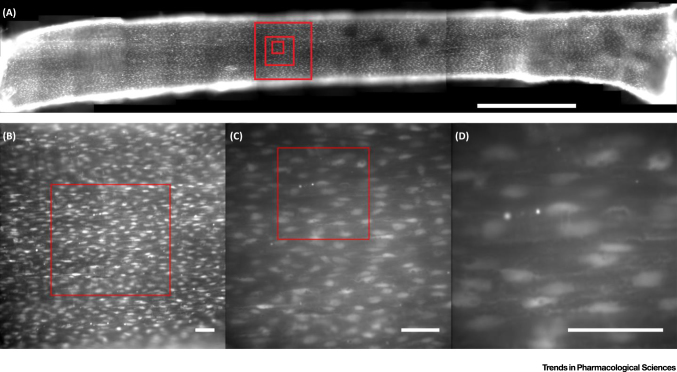
The Under-Appreciated Scale of the Endothelium. The endothelium is an extensive network of cells covering the entire cardiovascular system. (**A**) The endothelium of a cut-open second-order branch of a rat mesenteric artery (∼250 μm diameter). The endothelial cells have been loaded with the fluorescent Ca^2+^ indicator Cal-520/AM. The dye is loaded throughout the cells; however, the nuclear region of each endothelial cell appears brighter because it is thicker. This image shows approximately 5000 individual cells in a ∼7-mm length of artery. (A) is a 2 × 16 stitched image (20× magnification). Bar, 1 mm. (**B**–**D**) Single images taken at (B) 20×, (C) 40×, and (D) 100× magnification. Each region corresponds to the outlined box in the previous panel. Boxes corresponding to each of (B–D) are shown in (A). Bars, 50 μm.

While detection is difficult, coordination and consensus is a major challenge. In adults there are approximately 10 trillion (10^13^) endothelial cells [5], a value 100 times greater than the number of neurons in the brain [6]. Even on a local scale coordination is a mammoth task. In small and larger rat arteries there are ∼2000 cells per square millimetre (Figure 1 and Video S1 in the supplemental information online) whose behaviour must also be coordinated to regulate local output. The question arises of how the behaviours of individual cells are coordinated across such an extensive network to control vascular function.

It is often tempting to attribute coordinated complex sensing and behaviour patterns to design or master control. Yet there is no direct central control of the endothelium, no coordinating centre, and no dominant cells. No endothelial cells have the ‘big picture’ of cardiovascular function. How do endothelial cells coordinate responses to multiple sensory inputs to control vascular function? Presently, there is a lack of credible explanations for how, minimally, tens of thousands of cells that are distributed across just a few millimetres of the blood vessel, each of which is capable of instructing blood vessel function, act together to coordinate vascular behaviour, let alone throughout the entire system. Here we propose that the complex sensing behaviours of the endothelium do not come from a coordinated, uniform response of all cells to instructions imposed by a controller. There is no master controller and complex noisy instructions from chemical activators are received from many sources. Rather, we suggest each cell has a very limited local sensing ability and samples the environment slightly differently from neighbouring cells based on the receptor complement expressed. Each cell provides only a small element of the overall information and cannot itself resolve the complexity of all information presented. Communication of information among interconnected cells increases the endothelium’s community information and permits solutions to complex sensory problems to be derived from the endothelial collective. The system behaviour is a result of aggregate activities and interactions among components of the system from which a distributed problem-solving ability arises that is not explicitly described by the properties of the individual cells. The process is similar to the mechanisms operating in ‘swarm intelligence’.

### Emergent Behaviour and Swarm Intelligence

Swarm intelligence is the organised behaviour of large communities that occurs without a global organiser [7]. The concept of swarm intelligence took inspiration from the behaviour of social insects (e.g., ants, termites, bees) and swarming, flocking, and herding behaviours in vertebrates and has become a computational and behavioural metaphor in the solution of distributed problems. The characterising property of swarm intelligence is the ability of a group to act in an apparently intelligent and coordinated way in the absence of an external controller. In communities of animals, swarm intelligence is acknowledged to address and answer complex questions like solving the shortest route to a destination and ways to avoid predators. Significantly, swarm intelligence provides a problem-solving capacity that arises from the interactions of individual components each of which by itself has very limited abilities (i.e., the intelligence is derived from network interactions among individuals and between individuals and the environment). The underlying principles of swarm intelligence are now operational features designed into artificial intelligence and robotics. We propose that the endothelium’s sensory system uses several of the same principles.

Swarm-intelligent systems have the following typical properties: (i) the system comprises many individuals that belong to only a few phenotypes; (ii) the overall behaviour of the system results from the interactions of individuals with each other and with their environment – that is, the group behaviour self-organises; and (iii) the interactions among the individuals are based on simple rules that use only local information that the individuals exchange directly.

Below we outline some of the features of the endothelium that are shared with these properties and an example of how the endothelium uses this type of behaviour in sensing. We suggest that, acting as single cells, interpreting complex chemical environmental changes is a virtually insolvable problem, but as a collective the endothelium responds quickly and effectively to the environment. In this example the endothelial collective is able to rapidly distinguish signal and noise in a noisy chemical environment, determine the concentration on a range of fluctuating values, and process multiple simultaneous inputs.

## Property 1: The Endothelium Comprises Many Individuals that Belong to a Few Typologies

Endothelial cells throughout the vascular system are relatively uniform. However, there are some structural and functional heterogeneities between different anatomical regions of the cardiovascular system [8]. Structural heterogeneity arises mainly from variation in the organisation of cells. While endothelial cells are mainly organised with the long axis of the cell parallel with the direction of blood flow, at artery branch points the cells are less organised and the cells have multiple directions. The physiological significance of the arrangement at branch points (if any) is unclear but these sites are prone to the development of atherosclerotic plaques. Other arrangements of endothelial cells occur in various regions. The endothelium of arteries and veins forms a continuous, uninterrupted layer of cells held together by tight junctions. The endothelium of capillaries may be ‘continuous’ or ‘fenestrated’ (i.e., containing pores) or ‘discontinuous’ (large gaps) according to the needs of the underlying tissue. Vascular beds of coronary, pulmonary, splanchnic, and skeletal muscle comprise continuous non-fenestrated endothelial cells that are tightly connected to each other and form a restrictive barrier 8, 9. An extreme example of a continuous endothelium is the blood–brain barrier, where endothelial cells form an exceptionally impermeable barrier [10]. Fenestrated endothelium is characteristic of organs involved in filtration or secretion, which include exocrine and endocrine glands and gastric and intestinal mucosa. Fenestrated endothelia are characterised by the presence of pores of a regular size (∼70 nm). Organs such as the liver, kidney, and lymphatics have vascular systems with discontinuous and highly permeable endothelial layers that have gaps of various sizes [11] required for the high filtration rates in each of these tissues [12]. These various structural arrangements in endothelial cells are central to the permission or prevention of filtration in different parts of the cardiovascular system.

In addition to structural/morphological changes, there also is variation in the response of endothelial cells in vessels of different anatomical origin [13]. The differences in responsiveness arise because endothelial cells in various regions express different receptors to generate at times different responses to the same stimulus 14, 15. Certain classes of receptor are found in some regions but not in others. For example, endothelial cells in the aorta and the mesenteric and femoral artery were positively immunostained for angiotensin II whereas endothelial cells in the pulmonary artery [16] and renal artery [17] were not. Expression of VWF (a protein that promotes blood clotting) is higher in the endothelium of veins compared with arteries and in large vessels compared with capillaries 18, 19.

The variation in protein expression among endothelial cells and in structure among tissues helps explain the differences in regional endothelial behaviour, and presumably these differences arise because different regions are exposed to distinct chemical and mechanical stimuli. The environments in different parts of the cardiovascular system may vary considerably (e.g., blood flow rates, pressure, oxygen tension, metabolites, growth factors, cytokines). These environmental differences will provide triggers for the expression of various proteins.

### Variation in Endothelial Cells within Regions

Interestingly, different endothelial cell phenotypes also occur within segments of the same blood vessels and even between neighbouring endothelial cells. These cells are presumably exposed to an identical extracellular environment, so the stimuli for protein expression must be similar [20]. This local variation in phenotype is largely unexplained (see [21]). For example, a mosaic pattern of microdomains of VWF-positive and -negative endothelial cells occurs in the capillaries of many vascular beds and in the aorta 16, 21, 22. There is also heterogeneity in the distribution of angiotensin II immunostaining in neighbouring endothelial cells of the femoral mesenteric artery [16]. Acetylcholine (ACh)-evoked Ca^2+^ responses are larger at branches in the rat thoracic aorta than at nearby non-branch regions [23]. The reverse was true for histamine [23]. The sensitivity to histamine and ACh was not distributed evenly among neighbouring cells but arranged in ‘belts’ of high sensitivity that varied by ∼100-fold along the flow lines [23]. In studies of murine thoracic aorta endothelial cells, while most cells (82%) responded to ATP, large fractions of cells did not respond to ACh, bradykinin, or substance P [24].

Together these studies show that neighbouring regions of the endothelium appear to be specialised to detect particular chemical activators. However, while it is appreciated to exist, the underlying reason for the heterogeneity is unclear (see below). What physiological role is served by cells just a few microns apart, and that are presumably exposed to virtually identical microenvironments, being endowed with different functions as determined by protein expression? We propose below that this facility provides the endothelial collective with an ability to solve complex sensory problems and process multiple parallel signals (see Property 3 below).

## Property 2: The Overall Behaviour of the System Results from the Interactions of Individuals with Each Other and with Their Environment

There is a wealth of experimental evidence showing that interaction occurs among endothelial cells. Investigations of electrical coupling and dye diffusion between cells demonstrate that the endothelium behaves as a functional syncytium allowing electrical and chemical signals to pass from cell to cell.

The evidence is clear from the electrical resistance of the cells. When isolated from the vascular wall, the input resistance of single isolated endothelial cells is high (tens of giga-ohms). However, in intact tissue endothelial cells have an exceptionally low input resistance. The low electrical resistance suggests that the cells are in electrical continuity via a low-resistance pathway. The input resistance of endothelial cells in rat aorta has been measured at 26–64 MΩ 25, 26 suggesting a high degree of coupling. Lymphatic endothelial cells range from 19 to 72 MΩ [27] and guinea pig mesenteric arteriole endothelial cells are at 4.6 MΩ [28]. In endothelial tubes from the superior epigastric arteries of mice, input resistance was 41 MΩ [29], while in the endothelium of rat small pulmonary arteries the input resistance was 39 MΩ [30]. The low electrical resistance of various intact endothelia suggests that the cells are in electrical continuity with each other.

Electrical measurements have also shown that the preferred route of communication is among endothelial cells rather than from endothelial cells to the underlying smooth muscle cells (i.e., the low resistance arises from endothelial cell–cell coupling not endothelial to smooth muscle cell coupling). The electrical coupling between the endothelial layer and the smooth muscle cells is of a very much higher resistance (900 MΩ) [28] than that among endothelial cells.

In addition to electrical coupling, studies examining the movement of fluorescent probes among cells suggest that neighbouring endothelial cells are well coupled and may communicate via intercellular transmission of chemicals 25, 29, 31. For example, when injected into specific single cells, fluorescent probes spread to adjacent cells that had not been exposed to the fluorophore, presumably by junctional transfer 25, 29, 31. Interestingly, and in keeping with the measured high electrical resistance values measured between the endothelium and smooth muscle cells, the fluorophores did not spread to the smooth muscle from endothelial cells 25, 31. The diffusion of fluorophores among endothelial cells was blocked reversibly by gap junction blockers (carbenoxolone or 18β-glycyrrhetinic acid) [29]. Together these experiments suggest that there is good coupling among neighbouring endothelial cells.

Coupling between endothelial cells permits transfer of information between neighbouring cells via the diffusion of ions or messenger molecules [32]. Many extracellular activators evoke responses by inducing changes in intracellular Ca^2+^ concentration 33, 34, 35 (but see [36]). The spread of messengers among cells, such Ca^2+^ or IP_3_, may generate a spatial gradient of information that travels from cell to cell 37, 38, 39 carrying information (a ‘Ca^2+^ wave’). For example, in the microcirculation localised application of the IP_3_-mobilising agonist acetylcholine activates a Ca^2+^ wave that travelled distances in excess of 1 mm from the application site 40, 41.

In larger arteries Ca^2+^ wave propagation between endothelial cells is less clearly demonstrated. When preparations of intact, coupled endothelial cell networks are activated by defined concentrations of agonists, the resultant profile of endothelial Ca^2+^ signalling dynamics is complex (Video S2 in the supplemental information) both within and between cells. Within individual cells, whole-cell Ca^2+^ signals may appear as: (i) a single transient increase that declines to baseline level; (ii) multiple transients each returning to baseline; (iii) a large initial elevation followed by a smaller but sustained plateau phase; or (iv) a large initial elevation followed by oscillations on an elevated plateau phase. Each of these types of signal arises from the propagation of one or more Ca^2+^ waves within an individual cell rather than a uniform simultaneous increase throughout the entire cell.

Several studies of endothelial signalling appear to show these single-cell Ca^2+^ increases propagating to neighbouring endothelial cells. Two main lines of experimental evidence suggest that the Ca^2+^ rises in neighbouring cells are propagating signals, rather than independent Ca^2+^ increases oscillating slightly out of phase with each other [42]. In the first, gap junction inhibitors, used to prevent communication among cells, blocked signal transmission [43]. However, gap junction blockers (carbenoxolone, 18β-glycyrrhetinic acid, Gap27) have a broad spectrum of activity and many off-target effects that may inhibit Ca^2+^ signals independently of effects on their putative target 29, 42, 44. In the second main line of evidence, photo-uncaging of caged IP_3_ was used to activate specifically identified endothelial cells. On activation an increase in Ca^2+^ always occurred in the targeted cell. Propagation to additional cells occurred when multiple neighbouring cells were activated simultaneously [42]. These experiments establish that communication between neighbouring endothelial cells does occur and suggest that the endothelium is a network of interconnected cells.

The precise path of transmission of multicellular signals in the endothelium will be determined by the nature of the connections in the network. The network’s construction determines the sensitivity of the system and how resilient the signalling system is to disruption. The endothelium’s functional repertoire also relies on the structural architecture of connections. Significantly, the endothelium acts as both a distributed sensory system [42] and a conduit for rapid information transfer over significant distances (e.g., in conducted or ascending vasodilation 45, 46). These two features (sensing and rapid communication) of the network are not necessarily easily reconciled (see below) from within a fixed structure but are achieved nonetheless. An understanding of the organisation of the network is required to appreciate endothelial function.

In other systems several types of network have been characterised based on the number of connections between nodes (i.e., cells in the case of the endothelium; Box 1). Two extremes of network design are those that are completely regular structures and those that are completely random structures. In a completely regular network there are repeating patterns that occur within an unvarying lattice structure (meshes). In these regular lattices, the numbers of connections at each node are virtually identical (i.e., there is very low heterogeneity). The repeating pattern means that the network structure has low (zero) randomness; the probability of any two randomly chosen nodes being directly connected approaches zero as the size of the network increases. In this type of network structure, all nodes cooperate in the distribution of data and messages are propagated by ‘hopping’ from node to node to its destination.

Another type of network structure is the ‘random Erdos–Renyi (ER) network’ (Box 1). In this type of network, all nodes have (stochastically) roughly the same number of connections. Because of the similar number of connections at each node there is little clustering (i.e., nodes with larger-than-average numbers of connections) and the average path length between nodes is short. Removing any random node is likely to increase the mean shortest path length slightly but significantly.

Biological networks may lie somewhere between completely regular schemes and random structures; that is, biological networks do not have a homogeneous distribution of connections like that of random networks or regular lattices [47]. Indeed, the numbers of connections at node in a biological network may vary substantially. The varying numbers of connections at each node in biological networks give rise to the feature of ‘modularity’ [i.e., dense connections between groups of certain nodes (modules) and sparse connections between nodes in different modules]. One example of a network that may exhibit modularity is the ‘scale-free network’ (Box 1). In a scale-free network, there is a small but significant number of nodes with many connections; these nodes are referred to as hubs. There also is a tail of nodes with very few connections. Many intracellular signalling systems, such as protein–protein interactions and metabolic networks, use a scale-free network design [47]. Scale-free networks combine heterogeneity and randomness in connectivity and may have low or high modularity. This type of network can be robust because faults arising in the network are likely to occur at random sites and are likely to have minimal effect on performance. Even if one hub is affected, other hubs will take over. If a major hub is affected, the system will be reduced to a few connections only. Thus, an essential part of network functionality depends on the well-being of major hubs.

The network arrangement of endothelial cells is critical to vascular function but is unclear. Endothelial cells are fixed in physical space and the physical connections between cells are invariant; the arrangement is a mesh. Despite this fixed anatomy, and mesh cell arrangement, the number of functional communication routes in the endothelium is large and gives rise to multiple possible outputs ([48], see Figure 4). Even a casual glance at communication across the endothelium shows the complex varying paths on activation (Video S2). The functional organisation of the endothelial network thus shows dynamic connectivity despite being a static structural entity. The dynamic, changing paths of communication arise in part from the refractory time each cell has after the Ca^2+^ increase, which forces signals to take alternative routes or terminate.

**Figure 2:**
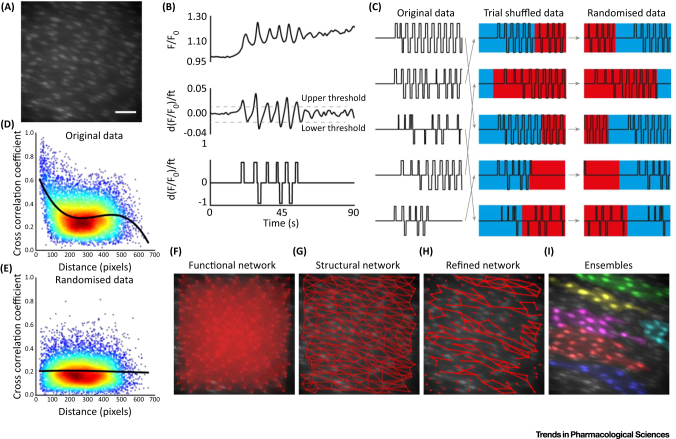
Network Analysis of Ca^2+^ Imaging Data Identifies Cellular Ensembles in Native Endothelium. (**A**) Ca^2+^ image of a field of endothelial cells (∼150) in an intact artery. Bar, 50 μm. (**B**) Processing of Ca^2+^ signals for correlation analysis. False correlations between Ca^2+^ signals (top), due to underlying trends, may be eliminated by taking the first derivative (middle). Artefacts due to amplitude fluctuations and sparse data (zero values) may then be eliminated by normalising the signal magnitude between zero and one (thresholded; bottom) and Gaussian noise added to prevent correlations arising between signals with low activity. (**C**) Trial shuffling and scrambling signals may be used to generate artificial randomised data for permutation testing. The thresholded data (left) are first deranged (randomly shuffled; middle) and then randomised by shuffling each signal (right) from a random time point. (**D**,**E**) Correlation as a function of distance between cells for original (D) and randomised (E) Ca^2+^ signals from an experiment imaging ∼150 cells. Repeating the randomised analysis (thousands of times) permits permutation testing. Comparing correlation values against the randomised distribution enables significant correlations to be identified. (**F**–**I**) Ca^2+^ image overlaid with: (F) functional network map showing all possible connections between cells; (G) structural network map showing all physical connections (e.g., gap junctions); (H) refined functional network map with significant correlations obtained by permutation testing and refined using the structural network; (**H**) colour-coded cell ensembles identified from Ca^2+^ imaging data.

One method of identifying the nature of the functional network properties and features of signal propagation and cooperativity is analysis of the latency between signals of endothelial cells [49]. Using this type of latency analysis, the extent of coordination of Ca^2+^ signals was found to be determined by the stimulus intensity and, critically, on the physical proximity between actively signalling cells [49]. In our own studies of endothelial Ca^2+^ signalling, we observed Ca^2+^ waves that seemingly propagate across clusters of cells in both pressurised and *en face* arteries. The propagation of Ca^2+^ waves among groups of cells was most apparent during prolonged (∼5–10 min) activation with an agonist (GSK1016790A, 30 nM) of transient receptor potential cation channel subfamily V member 4 (TRPV4) (Figure 2 and Video S3 in the supplemental information online) [48]. The coordinated nature of these waves suggests that the endothelium has a modular architecture. While these modular structures can be visually identified, characterisation of the network properties requires an extensive and quantitative description.

To characterise the network structure, sophisticated automated methods have recently been developed to analyse recordings of Ca^2+^ activity from large numbers of neurons 50, 51, 52. In these analyses cross-correlations are computed between every possible cell pair identified in recordings of Ca^2+^ activity. The cross-correlation provides a measure of similarity between two signals, accounting for any temporal delay that may result from the propagation of the signal. Cross-correlation analysis with robust statistical testing and filtering has been used to estimate functional connectivity within complex networks 50, 52 (Figure 2A–E). However, in their basic form these techniques ignore any underlying anatomical arrangements that may be required for true connectivity. A combined study of both functional and structural aspects is required for a complete understanding of any network. This may be achieved by refining functional connectivity estimates using structural information [53]. In the case of the endothelium, cells are physically connected only to directly abutting cells (Figure 2F). The physical structure of the network is therefore a relatively simple regular mesh. When statistically significant functional connectivity is integrated within this structural connectivity, highly connected communities of cells are identified (Figure 2G). These communities, or modules, correlate well with those identified manually from the propagation of Ca^2+^ waves (Figure 2H), confirming the propagation of signals to neighbouring cells. The resulting functional network is thus much more complex than a regular mesh network. Clusters of cells in close communication are apparent with more loosely connected communication to neighbouring regions via various routes (Figure 2). These features are consistent with a network with features of modularity; that is, dense connectivity within a module but sparse, weak extrinsic connections between modules. Thus the endothelium forms a hybrid mesh network with features of modularity.

An endothelial network with the property of modularity may be ideal in sensing stimuli [42]. However, networks with the high levels of local clustering associated with modularity are inefficient for the large-scale diffusion processes 54, 55 and directed information flow that are central to the communication in ascending vasodilation. How can large-scale diffusional processes be reconciled within a modular network? The answer may lie in the endothelium being able to operate as different types of network for different physiological events; for example, modularity used for sensing and a mesh network for transmission of signals in processes such as ascending vasodilation. The endothelial network thus appears to be flexible and the functional connectivity within the network may be reconfigured for specific tasks and physiological activities. How the network may modify its behaviour depending on signalling is unclear and requires further work. In neuronal networks task-dependent reconfiguration of functional connectivity of the network occurs by varying synaptic efficacy 56, 57. The information distributed may be determined by ‘predictive coding’, which constitutes a probabilistic model of incoming sensory information. The network may make predictions about the input, adjusting the connections between cells to deliver information in the most effective way 56, 57. It is unclear what the endothelial equivalent may be, but perhaps the extent of connections via gap junctions can be varied to provide different communication patterns in the endothelial network.

## Property 3: Interactions among the Individuals Are Based on Simple Rules that Exploit Only Local Information that the Individuals Exchange Directly

**Figure 3:**
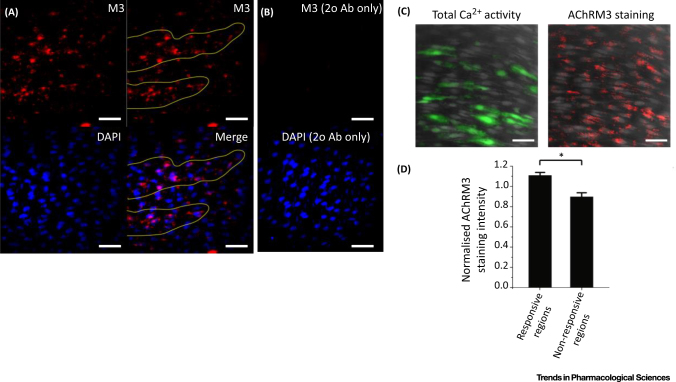
Distribution of Muscarinic Receptors. (**A**) Immunohistochemical localisation of endothelial M3 muscarinic acetylcholine receptors (AChRM3s) in the endothelium. Representative image (top left) illustrating that the AChRM3 distribution was not uniform across the endothelium but was more densely clustered in discrete regions (top right, yellow lines). In the same preparation, the nuclei of endothelial cells were labelled with DAPI (bottom left). An overlay (bottom right) of endothelial nuclei (blue) with AChRM3 (red) staining shows the clustered localisation of AChRM3s in particular regions of the endothelium (bottom right, yellow lines). (**B**) Negative control obtained by omitting anti-AChRM3 (top). DAPI loading (bottom) shows the positions of cell nuclei. (**C**) AChRM3s are increased in the most-sensitive cells activated by acetylcholine (ACh). Left: Total endothelial Ca^2+^ activity (green; evoked by 30-nM ACh) overlaid on cells (grey) in a carotid artery preparation. Right: Immunohistochemical localisation of endothelial AChRM3s (red) in the same field of endothelium from which the Ca^2+^ signals were obtained. Nuclei are shown in blue (DAPI staining). (**D**) Summary data showing that AChRM3s are more densely localised in ACh-sensitive regions of endothelium [i.e., green in (C)] compared with regions that are less sensitive (*n* = 3, *P* < 0.05). All bars, 50 μm. Reproduced from [42].

**Figure 4:**
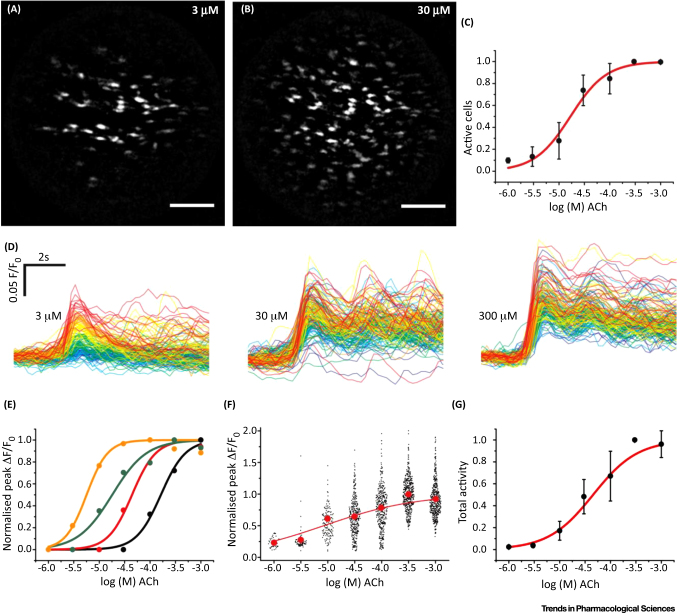
Graded Responses to Acetylcholine (Ach) Concentration. The number of cells activated increased with ACh concentration (**A**–**C**). Maximum-intensity projections (A,B) show the total number of cells activated at 3 μM (A) and 30 μM (B) ACh. Bars, 100 μm. As the concentration of ACh increases, more cells are activated. (C) Summarised data showing numbers of active cells (EC_50_ = 18.9 μM; 95% confidence interval, 7.25–49.4 μM; *n* = 3). (**D**) The amplitude of response in each cell also increased with the concentration of ACh. The responses to three illustrative concentrations (D) from the full concentration–response relationship are shown. The responses in each cell have been time aligned and colour coded based on the ACh sensitivity of the cells at the lowest ACh concentration (red, most sensitive; blue, least sensitive). As the concentration of ACh increases, the amplitude of the response increases. There is overlap in the response between 30 μM and 300 μM because of the position in the concentration–response relationship. (**E**) Representative concentration responses from four cells in one experiment showing a range of sensitivities to increasing ACh concentration. (**F**) Scatter plot of the overall responses from 445 cells from three arteries. The red dots plot the mean response at each concentration. The overall relationship appears flat because all responses at each concentration from separate arteries are shown. (**G**) Total endothelial response (EC_50_ = 42.7 μM; 95% confidence interval, 20.2–90.1 μM; *n* = 3) derived from the product of the number of active cells (C) and the mean response (F) at each concentration. Reproduced from [42].

Endothelial cells use extracellular receptors to constantly sample the local chemical and mechanical environment. However, as described for Property 1 above, the variation in expression of receptors means that the sensing capabilities of neighbouring cells to sample the environment are not the same. Each cell will sample the local chemical environment on the basis of its receptor complement, processing received information via network interactions among endothelial cells (Property 2). Our experiments have shown that, in addition to variation in the expression of different receptors on neighbouring cells, endothelial cells show heterogeneity in their expression of the same receptor on neighbouring cells (Figure 3) 42, 58. This variation in expression of the same receptors results in neighbouring endothelial cells being sensitive to different and limited ranges of concentrations of chemical activators (Figure 4) (see also [25]). Each cell’s response to the activator covers just over one order of concentration magnitude; that is, there is a steep concentration response over a single concentration order of magnitude. Each cell is therefore a highly sensitive detector of particular concentration ranges (Figure 4) and the sensitivity of each cell is matched to particular concentrations. However, the variation in sensitivity distributed among cells extends over three orders of concentration magnitude.

This arrangement – having cells that are highly sensitive to particular concentrations but variation in sensitivity among cells – solves one problem common in sensory systems; that is, how to create a highly sensitive detector that does not saturate at a relatively low-intensity stimulus. Endothelial cells of various sensitivities provides high sensitivity in the system and an enhanced concentration range over which the endothelium responds. The endothelium’s overall activity is a smoothly increasing response to increasing concentration of agonist (Figure 4G).

**Figure 5:**
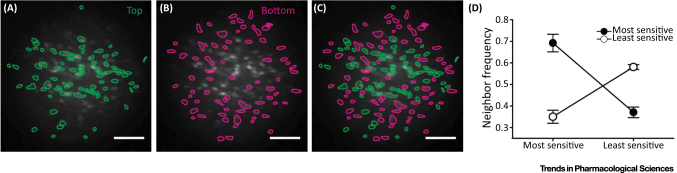
Endothelial Cells Cluster Based on Sensitivity. (**A**–**D**) Cells with comparable sensitivities to acetylcholine (Ach) are clustered. (A) The group of cells with the highest sensitivities to ACh (top 50%); (B) the group with the lowest sensitivities to ACh (bottom 50%); (C) a composite of (A) and (B). (D) Plots of neighbour frequency for high- and low-sensitivity cells normalised to the mean number of neighbours of all cells. Cells of a given sensitivity type (high or low) have significantly more neighbours of the same type. The ‘most-sensitive’ cells (top 50%) have more most-sensitive neighbours and fewer ‘least-sensitive’ (bottom 50%) neighbours. The least-sensitive cells (bottom 50%) have more least-sensitive neighbours and fewer most-sensitive (top 50%) neighbours. Bars, 100 μm. Reproduced from [42].

Significantly, endothelial cells with various sensitivities are not randomly distributed (Figure 5). Rather, cells with comparable sensitivities are clustered. The clustering creates domains of sensitivity. This clustering behaviour was previously reported in other studies 16, 21, 22, 23, 24 although the underlying physiological reason was unclear. Our results suggest the clustering provides a signal-coincidence detection system. When one cell alone was activated, there was limited communication to neighbouring cells [42]. However, when two or more adjacent cells were activated simultaneously pronounced communication occurred and propagating Ca^2+^ waves were transmitted to neighbouring cells [42]. The propagation provides secondary amplification and a means of communicating over distance [42]. The mechanisms for the propagation could involve the opening of gap junctions or the provision of sufficient IP_3_ by diffusion from the additive contributions of neighbouring cells to activate a propagating Ca^2+^ release.

The coincidence detection system may act to exclude noise by identifying when two or more cells are simultaneously active – an event unlikely to occur in the absence of an activator. Spontaneous, randomly occurring events (i.e., not agonist evoked) that occur in single cells are rarely transmitted to neighbouring cells. Thus, the endothelium recognises the spontaneous events as noise and does not generate a transmitted response. Detecting signals that are around basal values is a critical element in biological systems in complex environments. These signals will appear as small increases in concentration on a noisy baseline value. The mechanisms by which endothelial cells process small signals in noisy environments are not well understood. We propose that coincidence detection derived from the interactive behaviour of neighbouring cells improves the reliability and the precision of signal detection.

Endothelial cells decode chemical activators by using Ca^2+^ signals within cells. Coincidence detection may be facilitated by the periodic nature of the Ca^2+^ signals that occur within cells. In the presence of an activator, a Ca^2+^ increase occurs; Ca^2+^ then declines and the cell is ‘refractory’ for several seconds during which the store content and channels reset, and a Ca^2+^ increase may then occur again. The refractoriness and oscillations in the firing of Ca^2+^ spikes also may be instrumental in determining both the direction of wave propagation and the effectiveness of coincidence detection. Repeating signals within cells offer repeating opportunities for additional triggers to be recognised as ‘real’ signals. Ca^2+^ spiking synchrony may result in enhancement of signal detection and information transfer.

Importantly, the endothelium constantly processes multiple chemical signals each of which arrives sporadically and is just above the basal concentration value. As described above (Property 1), different cells express different receptors to detect various activators 16, 21, 22, 23, 24. In this way the endothelium may effectively process multiple parallel instructions (i.e., separate cells process multiple simultaneously arriving instructions). These features of the endothelium (cells of various sensitivities and distribution) create a robust detection system that matches cell to concentration and positions cells for maximum detection.

Together, the observations suggest several interesting sensing properties of the endothelium. (i) Sensing and control are fully distributed among numerous cells. (ii) Communication among the cells occurs in a highly localised way. (iii) System-level sensing is substantially greater than the sensing repertoire of any single individual cell. (iv) The system is organised so that each cell ‘pays close attention’ to the endothelial cells next to it. Each cell follows its own simple sensing rules based on sensitivity and the activity of its neighbours. These simple rules appear to be: (i) remain quiescent in the absence of an activator; (ii) respond when the concentration is in the correct range for the cell’s sensitivity; and (iii) respond when two or more neighbouring cells respond. These simple features constitute a sophisticated, wide-ranging detector in which the endothelium as a collective solves complex sensory problems that no single cell is aware exists.

## Swarm Sensing at Work

A central challenge of biology is to understand how systems detect information and respond to changes. Much of our current knowledge on detection is based on ensemble-averaged measurements from populations of cells. In the study of the endothelium there are a few common approaches. In one, endothelium-dependent regulation of the contraction of intact blood vessels is used as an indirect measure of endothelial function. These contraction measurements will usually involve the averaged activity of tens of thousands of endothelial cells contributing to the response. Another approach relies on cultured endothelial cells, which are often studied in some high-throughput screening of again, minimally, a few thousand cells. In each of these approaches, the endothelium is treated as a homogeneous population of cells that responds uniformly to each stimulus. Another approach to study the behaviour of the endothelium, rather than the averaged response of thousands of cells, is to examine one cell or a few cells that are taken to be representative of the entire population. Each approach has provided a great deal of important information on endothelial function but regards the entire population as homogeneous. Our results suggest that the endothelium is highly heterogeneous in behaviour and that heterogeneity is central to the effectiveness of the endothelium. The averaged, or ensemble, behaviour of the population may not represent the behaviour of any individual cell. Furthermore, the behaviour of any one cell may not be representative of the average response. The variance in single-cell responses is not a simple dispersion of variables around a mean but is crucial for the population response. The wide variance suggests that different cells are engaged in different tasks. The behaviour and interactions of neighbouring cells will result in complex feedback signalling. Activation of cells via propagating Ca^2+^ waves will alter the availability of a cell to respond to a different activator. There is currently virtually no information on how this feedback operates among cells and regulates Ca^2+^ signalling.

Studying large numbers of endothelial cells separately, we have found that sensing and control are fully distributed among numerous endothelial cells to provide a powerful, decentralised, sensitive, wide-ranging detection and control system. There are several advantages that emerge from this organisation of the endothelium.

(i) Parallel action: This is possible because endothelial cells express different receptors to allow different functions to be performed in different places at the same time.
(ii) Scalability: Because interactions in the endothelium involve only neighbouring individuals, the number of interactions tends not to grow with the overall number of individuals in the system. This means that a system can maintain its function while increasing its size without the need to redefine the way its parts interact. Scalability is interesting in the cardiovascular system as the system can easily increase in size (e.g., in angiogenesis) without the need to redefine any control structures.
(iii) Fault tolerance: The system comprises many interchangeable individuals capable of performing the sensing task – no single cell or hub controls the overall system behaviour. A failing individual can easily be substituted by another that is fully functioning. Fault tolerance is an inherent property of any system exhibiting swarm intelligence.

## Swarm Sensing and Pharmacological Targeting of the Endothelium

The sensing and control systems that we propose operate in the endothelium are inherently stable and fault tolerant. However, things do go wrong in cardiovascular disease. How do faults occur in a fault-resistant system and, from a therapeutic prospective, how can they be fixed? The first step in rational drug design is to identify the problem and target. Most approaches to the study of the underlying cellular problems in cardiovascular disease are population studies based on either large numbers of cultured cells or contraction/relaxation investigations in intact tissue. Implicit in these approaches is that the endothelium is a uniform population of cells. When changes in response are measured in cardiovascular disease with these approaches, the cells are usually considered to be uniformly affected. What we propose is that the cells are not uniform in function and as a result the problems in cardiovascular disease may be much more difficult to resolve than has been previously considered. High-sensitivity agonist sensing in the endothelium is achieved by clusters of cells that are tuned to particular agonist concentrations 42, 59. The cells in these clusters communicate with themselves and immediate neighbours. In cardiovascular disease, altered sensitivity to agonists may arise not because the sensitivity of all cells in the population has changed or even because the sensitivity of any cell has changed. Rather, altered sensitivity may arise because of a change in the distribution of cells that are otherwise completely normal. This change in distribution may significantly depress or enhance the function of the endothelium while the behaviour of each cell is unchanged. Alternatively, the communication between cells may be changed in cardiovascular disease or only certain sensitivity classes (not all) may be are altered.

Central to an understanding of the changes that occur in cardiovascular disease is one defining principle: the endothelial system (as in all complex systems) exists in a stable state that is achieved by a balance of multiple cell interactions and feedback processes. Alteration in the function of a key component (enzyme, ion channel) may trigger a change that forces the entire system into a new steady state, albeit one that is dysfunctional (i.e., cardiovascular disease). The new dysfunctional condition will again be maintained in a steady state by multiple altered interactions and feedbacks. However, because there will be multiple changes, the new steady state may not be easy to link to the initiating event. That is, the true underlying cause of a dysfunction in cardiovascular disease will be difficult to find. To establish the link between dysfunction and cause, full characterisation of the interactions and feedbacks of the system is required. The integrated sensing and communication properties of the endothelial network may need to be studied and understood in the way that communities of social insects or other animal societies have been studied in swarm-intelligent behaviour. It may be fruitless to attach any particular significance to changes in biomarkers/proteins (e.g., ion channels, enzymes) in cardiovascular disease. These changes may be a consequence rather than the cause of the condition. Furthermore, pharmacologically altering the behaviour of a biomarker/protein found to be altered in cardiovascular disease may not restore the system. These pharmacological agents may cause additional changes in overall function in ways that are difficult to predict because yet another new steady state could arise. The interactions and feedbacks that occur in complex systems may explain why the development of many, perhaps most, successful clinically used drugs has been through serendipity rather than rational drug design [60]. A successful approach to rational therapeutic development in any failing cell network that possesses the collective faculty of swarm intelligence will require an understanding of the vital resources that support the entire network’s structure and function and the impaired forces in the network’s self-defence that occur in cardiovascular disease.

## Concluding Remarks

The endothelium occupies a pivotal cardiovascular niche by interfacing blood vessels/tissue and blood supply. In its unique location, the endothelium uses an exquisite sensing ability to constantly monitor a wide range of extracellular chemicals that circulate in the blood supply or arrive from various cell types. In response to small changes in these chemicals, the endothelium alters vascular function to maintain cardiovascular homeostasis. In this review we have summarised current research showing that the endothelium is a collection of heterogeneous cells with various sensitivities to concentrations and pharmacological activators. We suggest that the heterogeneity is central to the exquisite sensing capability of the endothelium. Sensing, we propose, does not come from single cells acting uniformly but from the combined activity of a population of endothelial cells. Each cell has a limited, but distinctive, sensory capacity and shares information with neighbours so that sensing is distributed among cells. Communication of information among connected cells provides collective sensing that is substantially greater than the capabilities of any single cell. However, much research is still required to understand the roles of various sensing cells and the changes in the behaviour of the endothelium that characterise vascular disease. While there is significant data showing differences in sensitivity between various regions of the endothelium, including between neighbouring cells, the functional consequences in activities such as control of nitric oxide production, wound repair, or angiogenesis remain to be determined. Does heterogeneity contribute to function throughout the vascular system? In capillaries the number of neighbouring cells is significantly reduced compared with larger vessels. However, even in capillaries clear heterogeneity exists [21]. It will be particularly interesting to map specific signalling network pathways that link signal input and functional output (see Outstanding Questions). What interactions and feedback processes operate among cells to determine sensitivity and signal propagation? How does the network modify its behaviour and signal propagation depending on inputs and what interactions among cells determine whether short- or long-range communication occurs? Despite the unknowns, understanding the collective behaviour of the endothelium opens new opportunities for the appreciation of endothelial function and the translation of endothelial research into clinical vascular pharmacology, and the changes that occur in vascular disease and ageing of the cardiovascular system.

Outstanding Questions What are the endothelium’s sensitivity phenotypes?

What is the distribution of sensing cells?

What network pathways are used to communicate various stimuli?

How is the distribution of sensing cells and network communication systems changed in vascular disease?

## Figures and Tables

**Figure I:**
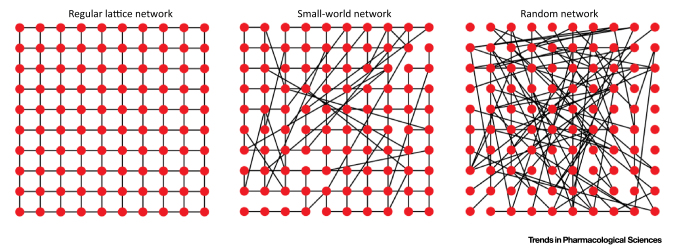
Network Structures. Schematic illustration of regular (left), small-world (middle), and random (right) networks. In the lattice network, each node is connected to its nearest neighbours. In this example the mean degree is slightly less than 4 because the network is not wrapped around on itself. In the small-world network, the edges of the regular lattice network have been randomly rewired with probability *P* such that long-range connections have emerged. In the random network, all edges have been randomly wired resulting in a network with little clustering.

## Box 1 Endothelial Networks

The structural properties of a network may limit or enhance overall system behaviour. To understand how structural properties may affect system performance, it is important to appreciate how components interact with one another. A system wiring diagram is used frequently for this purpose. The mathematical foundation for the study of such wiring diagrams is graph theory. A graph in this context is not a diagram of *x* versus *y* but a collection of elements (nodes) that may be connected structurally or functionally with each other via ‘edges’. Thus, a graph of a multicellular Ca^2+^ signalling network may be depicted as signalling links (edges; e.g., gap junctions) between interacting cells (nodes). The number of edges associated with each node is a property called node degree and the degree distribution is used to quantify the diversity of the entire network. Using these graphs the topology or architecture of the network may be characterised to determine various measures of connectivity [degree distribution, shortest path, diameter (maximum distance between any pair of nodes)], clustering/modularity, and robustness.

Classically, networks have been classified as being either regular or random. Regular networks are highly ordered structures in which each of *N* nodes is connected to *k* neighbouring nodes. The degree distribution of a regular network follows a delta-like function. Regular networks are thus an example of a homogeneous, ordered topology where the probability of any two randomly chosen nodes being directly connected approaches zero as the number of nodes increases towards infinity. An example of a regular network is the square lattice network (Figure I). These networks have a high degree of clustering and large path lengths, but are particularly robust because of the large number of possible paths between any two nodes such that damage to one node will not significantly alter information flow. In contrast to the regular network, random networks are highly disordered. A random network comprises *N* nodes connected to each other with probability P (Figure I). A random network is characterised by a Poisson-like degree distribution, a short average path length, a low clustering coefficient, and hubs (nodes with more than the average number of connections) do not occur [61].

Most networks encountered in nature do not display the ordered structure of a regular network but neither do they exhibit the narrow distribution of degrees displayed by random networks. Biological networks lie somewhere between completely regular and random structures; that is, biological networks often exhibit significant numbers of highly connected nodes and the number of connections at each node may vary substantially.

One network that combines some features of both regular and random topologies is the ‘small-world’ network. In small-world networks most nodes are not neighbours but all can be reached from every other node with a small number of ‘hops’. A specific formulation of the small-world network aimed to describe the transition from a regular lattice to a random graph [62]. This so-called Watts–Strogatz small-world network model may be realised by randomly rewiring each edge of a regular lattice network with probability *P* (Figure I). The resultant graph possesses high clustering due to dense local connections and short average paths across the whole network with occasional long links. These long links may be achieved in the endothelium by the release of diffusible substances such as nitric oxide or prostaglandin. Such small-world network architectures may be found in neural networks in the brain [63] or in large metabolic networks [64]. As it is an extension of the random network model, the Watts–Strogatz small-world network model also exhibits a Poisson-like degree distribution and nodes with a large degree distribution are absent.

Rather than being a Poisson function, the degree distribution of most biological networks is highly skewed and the number of nodes with any given degree is described by a power law. Such networks are termed ‘scale-free’ by analogy with fractals and phase transitions, which lack a characteristic scale 65, 66. As a result of the power-law distribution of node connections, a scale-free network possesses a small number of highly connected nodes (hubs) that dominate the network topology. Similarly, there are few nodes with very few connections. The advantage of scale-free networks is their resistance to random failure of, or attacks on, random nodes. However, scale-free networks are particularly vulnerable to major hubs [67].

Alt-text: Box 1

## References

[1] Sandison M.E. (2016). The transition of smooth muscle cells from a contractile to a migratory, phagocytic phenotype: direct demonstration of phenotypic modulation. J. Physiol..

[2] Pober J.S., Sessa W.C. (2007). Evolving functions of endothelial cells in inflammation. Nat. Rev. Immunol..

[3] Semenza G.L. (2010). Vascular responses to hypoxia and ischemia. Arterioscler. Thromb. Vasc. Biol..

[4] Fraisl P. (2009). Regulation of angiogenesis by oxygen and metabolism. Dev. Cell.

[5] Galley H.F., Webster N.R. (2004). Physiology of the endothelium. Br. J. Anaesth..

[6] Azevedo F.A. (2009). Equal numbers of neuronal and nonneuronal cells make the human brain an isometrically scaled-up primate brain. J. Comp. Neurol..

[7] Garnier S. (2007). The biological principles of swarm intelligence. Swarm Intell..

[8] Aird W.C. (2007). Phenotypic heterogeneity of the endothelium: I. Structure, function, and mechanisms. Circ. Res..

[9] Stevens T. (2008). Lung vascular cell heterogeneity: endothelium, smooth muscle, and fibroblasts. Proc. Am. Thorac. Soc..

[10] Reese T.S., Karnovsky M.J. (1967). Fine structural localization of a blood–brain barrier to exogenous peroxidase. J. Cell Biol..

[11] Mehta D., Malik A.B. (2006). Signaling mechanisms regulating endothelial permeability. Physiol. Rev..

[12] Wisse E. (1970). An electron microscopic study of the fenestrated endothelial lining of rat liver sinusoids. J. Ultrastruct. Res..

[13] Vanhoutte P.M., Miller V.M. (1985). Heterogeneity of endothelium-dependent responses in mammalian blood vessels. J. Cardiovasc. Pharmacol..

[14] Turner R.R. (1987). Endothelial cell phenotypic diversity. *In situ* demonstration of immunologic and enzymatic heterogeneity that correlates with specific morphologic subtypes. Am. J. Clin. Pathol..

[15] Page C. (1992). Antigenic heterogeneity of vascular endothelium. Am. J. Pathol..

[16] Tomlinson A. (1991). An immunohistochemical study of endothelial cell heterogeneity in the rat: observations in “*en face*” Hautchen preparations. Cell Tissue Res..

[17] Lincoln J. (1990). Localization of vasopressin, serotonin and angiotensin II in endothelial cells of the renal and mesenteric arteries of the rat. Cell Tissue Res..

[18] Yamamoto K. (1998). Tissue distribution and regulation of murine von Willebrand factor gene expression *in vivo*. Blood.

[19] Aird W.C. (1997). Vascular bed-specific expression of an endothelial cell gene is programmed by the tissue microenvironment. J. Cell Biol..

[20] Aird W.C. (2005). Spatial and temporal dynamics of the endothelium. J. Thromb. Haemost..

[21] Yuan L. (2016). A role of stochastic phenotype switching in generating mosaic endothelial cell heterogeneity. Nat. Commun..

[22] Senis Y.A. (1996). Changes in the pattern of distribution of von Willebrand factor in rat aortic endothelial cells following thrombin generation *in vivo*. Br. J. Haematol..

[23] Huang T.Y. (2000). Heterogeneity of [Ca^2+^]_i_ signaling in intact rat aortic endothelium. FASEB J..

[24] Marie I., Beny J.L. (2002). Calcium imaging of murine thoracic aorta endothelium by confocal microscopy reveals inhomogeneous distribution of endothelial cells responding to vasodilator agents. J. Vasc. Res..

[25] Carter T.D., Ogden D. (1994). Acetylcholine-stimulated changes of membrane potential and intracellular Ca^2+^ concentration recorded in endothelial cells *in situ* in the isolated rat aorta. Pflugers Arch..

[26] Marchenko S.M., Sage S.O. (1993). Electrical properties of resting and acetylcholine-stimulated endothelium in intact rat aorta. J. Physiol..

[27] von der Weid P.Y., Van Helden D.F. (1997). Functional electrical properties of the endothelium in lymphatic vessels of the guinea-pig mesentery. J. Physiol..

[28] Yamamoto Y. (2001). Intercellular electrical communication among smooth muscle and endothelial cells in guinea-pig mesenteric arterioles. J. Physiol..

[29] Behringer E.J. (2012). Electrical conduction along endothelial cell tubes from mouse feed arteries: confounding actions of glycyrrhetinic acid derivatives. Br. J. Pharmacol..

[30] Olschewski A. (2001). Basic electrical properties of *in situ* endothelial cells of small pulmonary arteries during postnatal development. Am. J. Respir. Cell Mol. Biol..

[31] Beny J.L., Gribi F. (1989). Dye and electrical coupling of endothelial cells *in situ*. Tissue Cell.

[32] Schmidt K. (2016). Communication through gap junctions in the endothelium. Adv. Pharmacol..

[33] Tran Q.K. (2000). Calcium signalling in endothelial cells. Cardiovasc. Res..

[34] Dora K.A. (1997). Elevation of intracellular calcium in smooth muscle causes endothelial cell generation of NO in arterioles. Proc. Natl Acad. Sci. U. S. A..

[35] Noren D.P. (2016). Endothelial cells decode VEGF-mediated Ca^2+^ signaling patterns to produce distinct functional responses. Sci. Signal..

[36] Stolwijk J.A. (2016). Calcium signaling is dispensable for receptor regulation of endothelial barrier function. J. Biol. Chem..

[37] Olson M.L. (2010). Mitochondrial Ca^2+^ uptake increases Ca^2+^ release from inositol 1,4,5-trisphosphate receptor clusters in smooth muscle cells. J. Biol. Chem..

[38] Bradley K.N. (2003). Cyclic ADP-ribose increases Ca^2+^ removal in smooth muscle. J. Cell Sci..

[39] McCarron J.G. (2010). Agonist-evoked Ca^2+^ wave progression requires Ca^2+^ and IP_3_. J. Cell Physiol..

[40] Tallini Y.N. (2007). Propagated endothelial Ca^2+^ waves and arteriolar dilation *in vivo*: measurements in Cx40BAC GCaMP2 transgenic mice. Circ. Res..

[41] Bagher P. (2011). Intravital macrozoom imaging and automated analysis of endothelial cell calcium signals coincident with arteriolar dilation in Cx40^BAC^–GCaMP2 transgenic mice. Microcirculation.

[42] Wilson C. (2016). Clusters of specialized detector cells provide sensitive and high fidelity receptor signaling in intact endothelium. FASEB J..

[43] Emerson G.G., Segal S.S. (2000). Electrical coupling between endothelial cells and smooth muscle cells in hamster feed arteries: role in vasomotor control. Circ. Res..

[44] Tare M. (2002). Glycyrrhetinic derivatives inhibit hyperpolarization in endothelial cells of guinea pig and rat arteries. Am. J. Physiol. Heart Circ. Physiol..

[45] Bagher P., Segal S.S. (2011). Regulation of blood flow in the microcirculation: role of conducted vasodilation. Acta Physiol..

[46] Murrant C.L., Sarelius I.H. (2015). Local control of blood flow during active hyperaemia: what kinds of integration are important?. J. Physiol..

[47] Albert R. (2005). Scale-free networks in cell biology. J. Cell Sci..

[48] Wilson C. (2016). Acetylcholine released by endothelial cells facilitates flow-mediated dilatation. J. Physiol..

[49] Socha M.J. (2012). Coordination of intercellular Ca^2+^ signaling in endothelial cell tubes of mouse resistance arteries. Microcirculation.

[50] Smedler E. (2014). Network analysis of time-lapse microscopy recordings. Front. Neural Circuits.

[51] Malmersjo S. (2013). Small-world networks of spontaneous Ca^2+^ activity. Commun. Integr. Biol..

[52] Malmersjo S. (2013). Neural progenitors organize in small-world networks to promote cell proliferation. Proc. Natl Acad. Sci. U. S. A..

[53] Ullo S. (2014). Functional connectivity estimation over large networks at cellular resolution based on electrophysiological recordings and structural prior. Front. Neuroanat..

[54] Granovetter M.S. (1973). The strength of weak ties. Am. J. Sociol..

[55] Watts D.J. (2003). Small Worlds: The Dynamics of Networks between Order and Randomness.

[56] Friston K.J. (2010). The free-energy principle: a unified brain theory?. Nat. Rev. Neurosci..

[57] Park H.J., Friston K.J. (2013). Structural and functional brain networks: from connections to cognition. Science.

[58] Wilson C. (2015). Pressure-dependent regulation of Ca^2+^ signaling in the vascular endothelium. J. Physiol..

[59] Wilson C. (2017). Advancing age decreases pressure-sensitive modulation of calcium signaling in the endothelium of intact and pressurized arteries. J. Vasc. Res..

[60] Prasad S. (2016). Serendipity in cancer drug discovery: rational or coincidence?. Trends Pharmacol. Sci..

[61] Erdos P., Renyi A. (1960). On the evolution of random graphs. Bull. Int. Stat. Inst..

[62] Watts D.J., Strogatz S.H. (1998). Collective dynamics of ‘small-world’ networks. Nature.

[63] Stephan K.E. (2000). Computational analysis of functional connectivity between areas of primate cerebral cortex. Philos. Trans. R. Soc. Lond. B Biol. Sci..

[64] Wagner A., Fell D.A. (2001). The small world inside large metabolic networks. Proc. Biol. Sci..

[65] Albert R. (1999). Internet – diameter of the world-wide web. Nature.

[66] Barabasi A.L., Albert R. (1999). Emergence of scaling in random networks. Science.

[67] Albert R. (2000). Error and attack tolerance of complex networks. Nature.

