# Supplementary figures and images for: The Endothelium Solves Problems That Endothelial Cells Do Not Know Exist
Source: Trends Pharmacol Sci. 2017 Apr;38(4):322–38. doi: 10.1016/j.tips.2017.01.008 (PMC5381697; doi:10.1016/j.tips.2017.01.008)

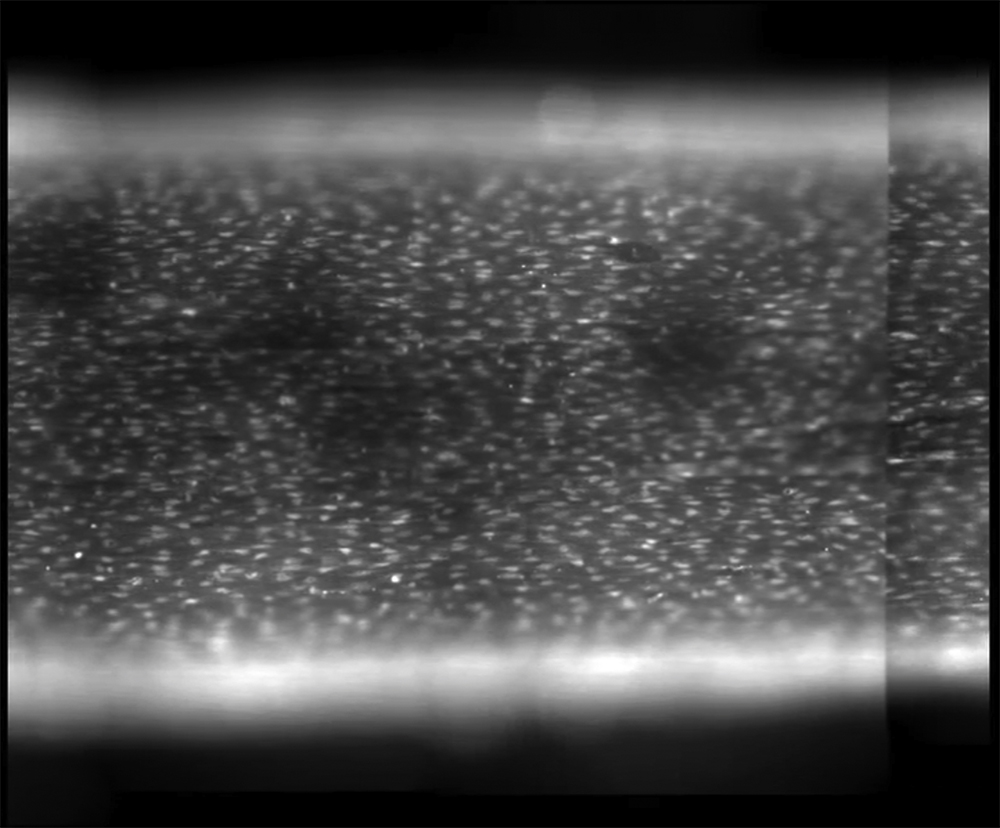

Supplement: Supplementary file 1 [file mmc1.jpg]

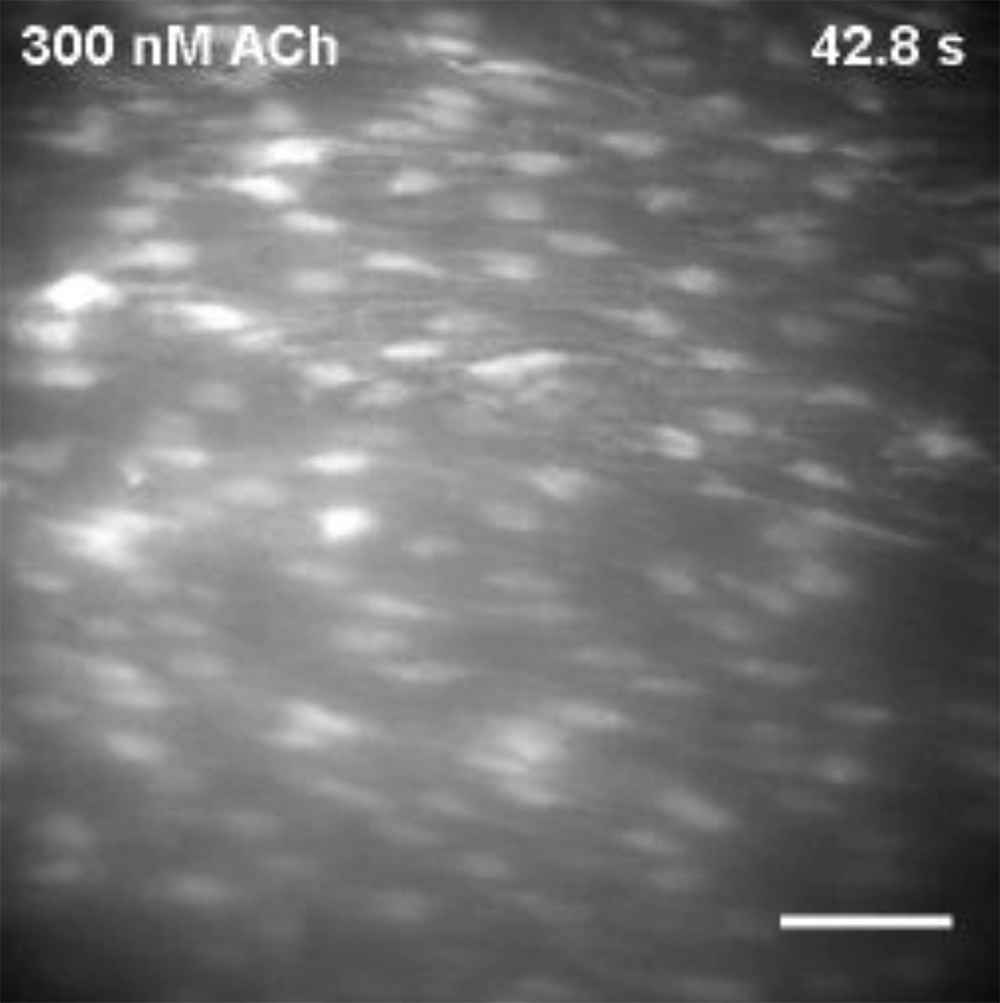

Supplement: Supplementary file 2 [file mmc2.jpg]

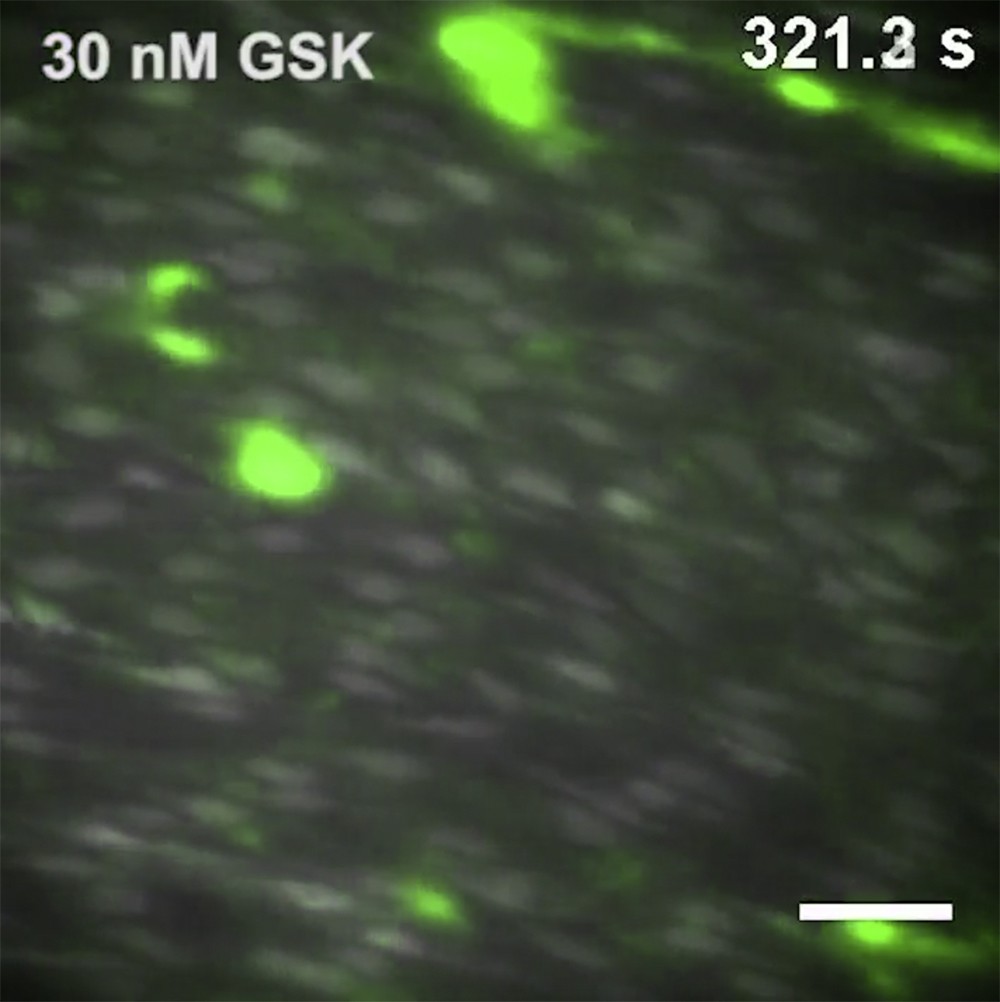

Supplement: Supplementary file 3 [file mmc3.jpg]
